# System-wide identification of myeloid markers of TB disease and HIV-induced reactivation in the macaque model of Mtb infection and Mtb/SIV co-infection

**DOI:** 10.3389/fimmu.2022.777733

**Published:** 2022-10-05

**Authors:** Maya Gough, Dhiraj K. Singh, Bindu Singh, Deepak Kaushal, Smriti Mehra

**Affiliations:** Southwest National Primate Research Center, Texas Biomedical Research Institute, San Antonio, TX, United States

**Keywords:** myeloid, tuberculosis, reactivation, active TB, latent TB, SIV, macrophage, nonhuman primate

## Abstract

*Mycobacterium tuberculosis* (*Mtb*) has developed specialized mechanisms to parasitize its host cell, the macrophage. These mechanisms allow it to overcome killing by oxidative burst and persist in the wake of an inflammatory response. *Mtb* infection in the majority of those exposed is controlled in an asymptomatic form referred to as latent tuberculosis infection (LTBI). HIV is a well-known catalyst of reactivation of LTBI to active TB infection (ATB). Through the use of nonhuman primates (NHPs) co-infected with *Mtb* and Simian Immunodeficiency Virus (*Mtb*/SIV), we are able to simulate human progression of TB/AIDS comorbidity. The advantage of NHP models is that they recapitulate the breadth of human TB outcomes, including immune control of infection, and loss of this control due to SIV co-infection. Identifying correlates of immune control of infection is important for both vaccine and therapeutics development. Using macaques infected with *Mtb* or *Mtb*/SIV and with different clinical outcomes we attempted to identify signatures between those that progress to active infection after SIV challenge (reactivators) and those that control the infection (non-reactivators). We particularly focused on pathways relevant to myeloid origin cells such as macrophages, as these innate immunocytes have an important contribution to the initial control or the lack thereof, following *Mtb* infection. Using bacterial burden, C-reactive protein (CRP), and other clinical indicators of disease severity as a guide, we were able to establish gene signatures of host disease state and progression. In addition to gene signatures, clustering algorithms were used to differentiate between host disease states and identify relationships between genes. This allowed us to identify clusters of genes which exhibited differential expression profiles between the three groups of macaques: ATB, LTBI and *Mtb*/SIV. The gene signatures were associated with pathways relevant to apoptosis, ATP production, phagocytosis, cell migration, and Type I interferon (IFN), which are related to macrophage function. Our results suggest novel macrophage functions that may play roles in the control of *Mtb* infection with and without co-infection with SIV. These results particularly point towards an interplay between Type I IFN signaling and IFN-γ signaling, and the resulting impact on lung macrophages as an important determinant of progression to TB.

## Introduction

Tuberculosis (TB), caused by *Mycobacterium tuberculosis* (*Mtb*), is the most prevalent opportunistic disease in human immunodeficiency virus (1)-infected individuals globally. Worldwide, TB coinfection accounts for >30% of total HIV associated deaths (2). In 2020, 214,000 people who had both TB and HIV were estimated to have died. This was in addition to the 1.3 million people who died from TB alone that year (2). Understanding the interaction between these two deadly infections is therefore, a critical research and public health priority (3, 4). *Mtb* infection can have a spectrum of different outcomes inside a human host, ranging from a life-long asymptomatic infection termed latent tuberculosis infection (LTBI) to spontaneous reactivation of LTBI. HIV co-infection generates further heterogeneity in the outcome of *Mtb* infection.

*Mtb predominantly spreads by aerosol transmission and lungs are the primary site of infection*. Alveolar macrophages are the first immunocytes infected by Mtb. However numerous reports have established impaired ability of AMs to elicit a potent antibacterial response. *Mtb* has developed specialized mechanisms to protect against the different macrophage-mediated pathways of pathogen clearance. These include induction of a multitude of anti-oxidants under the influence of the transcription factor SigH (5, 6), interference with phago-lysosomal fusion and acidification (7, 8) and with the induction of IFN-gamma (IFN-γ) responses (9). It has recently been argued that two different types of macrophages in the lungs, alveolar, and interstitial, exhibit different responses and killing capacity in response to *Mtb* infection (10). The concept of heterogeneity of macrophage responses to *Mtb* has been further broadened by the use of single-cell RNAseq (11) approaches combined with innovative fluorescent reporter strains to identify bystander macrophages from those productively infected with *Mtb* (12). Greater insights are however needed into how diverse subsets of macrophages present in the lung respond to *Mtb* infection, and the resulting ability of the host to control infection.

We have developed infection models of *Mtb*, and *Mtb*/SIV co-infection, in susceptible rhesus macaques (RMs) (13). Upon exposure with a high-dose of *Mtb* CDC1551 strain *via* the inhalation route as aerosols, RM develop active TB characterized by high levels of bacterial replication in the lung granulomas. Bacilli are also readily detected in the alveolus, and ample clinical and radiological evidence of TB is present (14). At the time of euthanasia, the lungs of these animals are characterized by the presence of primarily necrotic granulomas, although presentations in non-alveolar tissues like lymph nodes, spleen, liver, kidney, brain etc. are possible (14). Necrosis is strongly associated with hypoxia, which profoundly impacts both the ability of the bacilli to replicate in macrophages and the ability of the adaptive immune response to eradicate the pathogen, thus permitting persistence (15). Upon exposure to low-dose *Mtb* CDC1551, some RMs develop immunological signs of infection (tuberculin skin test positivity or T cell responses to *Mtb* antigen in immunological assays), but without clinical and radiological signs of TB. At necropsy, these animals are typically characterized by the presence of a solitary, or a few fibrotic, but centrally necrotic granulomas, with very low bacterial burden. However, these bacilli are persistent, since co-infection with Simian Immunodeficiency Virus (SIV; in this instance SIVmac239) leads to acute viral infection and reactivation of *Mtb* infection. These RM are characterized by the formation of newer granulomas and dissemination of both bacilli and granulomatous inflammation to extrathoracic sites (16, 17). Importantly, both *Mtb* and SIV co-localize in lung macrophages (10). This gives rise to the hypothesis that SIV co-infection of macrophages may impair *Mtb*-specific responses leading to reactivation. Indeed, experiments in this model suggest that the mere depletion of CD4^+^ T cells is not sufficient to cause reactivation (18). We have postulated that chronic immune activation due to SIV (1) co-infection, mediates macrophage turnover and immune dysfunction, resulting in reactivation (19). Consistent with this, initiation of highly active anti-retroviral therapy (HAART) at a time when signs of macrophage turnover are apparent fails to protect *Mtb*/SIV co-infected RM from reactivation, despite impressive control of viral replication and reconstitution of CD4+ T cells (20). Initiation of ART at earlier time-points is more beneficial, further suggesting that macrophage turnover is the critical determinant of lung-specific chronic immune activation during *Mtb*/SIV co-infection which causes reactivation (1).

We compared system-wide transcriptional responses in the lungs of RMs to *Mtb* infection (leading to either progression to active TB – ATB; or immune-mediated control of infection – LTBI). Also included in the study were samples from RMs with *Mtb*/SIV co-infection which mostly results in reactivation of LTBI. Banked samples were selected from macaques with ATB, LTBI, and *Mtb* /SIV co-infection for our cohorts. Naïve samples were collected from opportunistic necropsies of TB naïve RMs. Samples were profiled by RM whole-genome DNA microarray and analyzed through a data analytics pipeline. We attempted to identify patterns intrinsic and unique to LTBI, ATB, and *Mtb*/SIV co-infection and particularly focused on macrophage responses. Our results suggest that it may be possible to identify peripheral markers and gene regulation indicative of reactivation and non-reactivation. This study represents a preliminary step in this direction.

## Methods

### Data acquisition

Animals were chosen if they were experimentally infected with 10-25 Colony Forming Units (CFU) of *Mtb* (CDC1551 strain) or co-infected with *Mtb* (CDC1551)/SIV (SIVmac239) during 2011-2014 but were not treated with any antibiotics. Co-infection with SIV was always performed at 9 weeks post-*Mtb* infection, and with 300 TCID50 virus using the intravenous route, as described in our previous publications (1, 16–18, 20, 21). CD4 depletion in blood and BAL along with plasma viral load was used to confirm productive SIV infection. All co-infected animals devoid of any signs of TB disease at the time of co-infection were deemed to have developed LTBI. Lung samples from co-infected animals were obtained 4-8 weeks post-SIV infection. Adult (3-12 year old) animals of both sexes were used insofar as possible. Methods for quantification of lung bacterial burdens (CFU/gm) have been described earlier (15–18, 20, 22, 23). Briefly, Tissues were collected, homogenized and plated onto 7H11 Middlebrook plates containing 10% OADC supplement at necropsy. CFUs were determined per gram of tissue, with four sections of pulmonary tissue collected to represent every lung lobe. Bacterial burden, viral load, and immunologic response were determinants for classification to either active (ATB, n=16), latent (LTBI, n=11), *Mtb*/SIV co-infected (*Mtb*/SIV, n=8), and naïve control (CTRL, n=6) groups. Through the use of serum chemistry, CRP values, and Albumin/Globulin (A/G) ratios were obtained (24). Blood cell percentages for monocytes, lymphocytes, and neutrophils were also quantified. Post-infection minimal and maximal values observed throughout the experiment until necropsy were denoted as bottom and peak, respectively, and used throughout the data processing. Gene expression in lung tissue was enumerated through DNA microarray as Log_2_ fold change, as previously described. Briefly, experimental and CTRL lung RNA samples were Cy5 and Cy3 labelled respectively and the resulting cDNA hybridized to Agilent 4x44k RM microarrays. Scanned.gpr files were analyzed in Spotfire DecisionSite package, using Lowess normalization, averaging of duplicate spots, and removal of unreliable data, as previously described (25, 26), resulting in a robust DNA microarray dataset. Raw files from this analysis will be deposited in Gene Expression Omnibus repository and can be accessed using the accession GPL10183.

### Dimensionality reduction and difference analysis

Initial dimensionality reduction of the robust DNA microarray dataset was performed through use of Fselector function in R programming language with RStudio as interface and 41 genes of interest (GOI) were selected using this package (http://www.rstudio.com/). Welch’s t tests and ordinary one-way ANOVA were performed to compare CTRL (n=6), ATB (n=16), LTBI (n=11), and *Mtb*/SIV (n=8) datasets. T tests were run on CFU/gm and (CFU/gm)log_10_ counts obtained from lung at endpoint and CRP values. A/G ratio, Lymphocyte, Monocyte, and Neutrophil percentages at pre-infection, peak, bottom, and endpoint were compared between all groups. Differential gene expression was also determined. Welch’s t tests were performed using R with RStudio interface and ordinary one-way ANOVA using GraphPad Prism version 8.0.0 (https://www.graphpad.com/). Bonferroni correction for multiple comparisons was applied and p-value thresholds were adjusted accordingly for respective comparisons.

### Correlation

Using R and the Excel extension StatPlus Pro we performed principal component analysis (PCA) (http://www.rstudio.com/). For PCA clustering we utilized an aggregate dataset of all groups, unlike correlation analysis which was performed on group delineated datasets. Analysis was performed on animal groups on an aggregate of all genes or on GOIs on an aggregate of all animals, to further expound on the relationships within each animal group and between GOIs. Pearson correlation analysis was performed using R. CTRL, LTBI, ATB, and *Mtb*/SIV datasets were analyzed. To fully demonstrate quantifiable differences between host disease states, analysis was performed within each respective group CTRL, LTBI, ATB, or *Mtb*/SIV, rather than on an aggregate dataset. We observed distinct correlative patterns that were present regardless of the level of bacterial burden and correlative patterns isolated to individual disease states.

### Cluster analysis

iDEP.94 (integrated web application for differential expression and pathway analysis) was used to perform K-means clustering and gene enrichment analyses (27). Due to the nature of the algorithm all missing data points were replaced with a value close to zero, 1e-15, for K means cluster analysis only. Gene Ontology (GO) terms that were enriched in the Genes of Interest (GOIs) were identified using DAVID Bioinformatics Resource (https://david.ncifcrf.gov/) (28, 29).

#### Validation of transcriptomics results by immunohistochemistry and multilabel confocal microscopy imaging

To validate the findings originating from microarray analysis, multilabel immuno-histochemistry was performed on formalin fixed paraffin embedded (FFPE) sections of lungs derived from RMs with ATB or LTBI, as described previously (11, 30). The lung sections were stained for macrophages with anti-CD68 (Thermo Fischer Scientific, Cat no #MA5-13324) and anti-MX1 (Thermo Fischer Scientific, Cat no #PA5-22101) antibodies to validate the *in-vivo* expression of these markers in lung tissue. DAPI (Thermo Fischer Scientific, Cat no #D1306) was used to stain nuclei. Images were captured using Zeiss Axio ScanZ1 and Zeiss LSM-800 confocal microscope and analysis was done using Zeiss Zen 3.6 (blue edition).

## Results

### Bacterial burden

**Figure 1:**
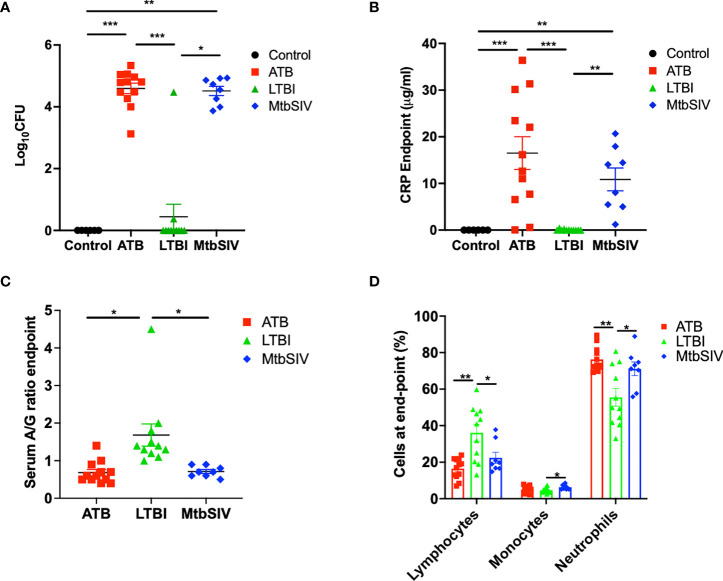
Clinical metrics at endpoint. At necropsy colony forming units(log_10_) are measured in the lung **(A)** and serum C-reactive protein **(B)**, serum albumin-to-globulin ratio **(C)**, percentage of cells **(D)** are measured in the periphery through the blood. For CFU **(A)** and CRP **(B)** we compared all four cohorts, control (black), ATB(red), LTBI (green), and *Mtb*/SIV (blue). Serum albumin and globin **(C)** and cells in the periphery measured through flow **(D)** cytometry. Data is shown for control (n=6), ATB (n=12), LTBI (n=11), and *Mtb*/SIV (n=8). Error bars represent Mean ± SEM. (*P value of ≤ 0.01; **P value of ≤ 0.001; ***P value of <0.0001).

*Mtb* lung CFU burden strongly correlates with host TB disease state and predicts the outcome of experimental infections in susceptible RM (10, 15–18, 22, 23, 25, 31–33). We have accumulated data from RMs which were either latently infected (LTBI, n=11), actively infected (ATB, n=16), or coinfected (*Mtb*/SIV, n=8); naïve samples were also collected from control animals (CTRL, n=6). NHPs were infected with *Mtb* only or *Mtb* followed by SIV nine weeks later, as described earlier (1, 16–18, 20, 21), during 2011-2014, and endpoint lung bacterial burdens were assessed. The lung bacterial burden for the ATB and LTBI groups were significantly different, though differences in CFU were not significant between LTBI and CTRL (**Figure 1A**, **Table 1**). Bacterial burdens in the ATB and *Mtb*/SIV groups were significantly higher than CTRL and LTBI, as previously described (17, 18), but not significantly different from each other (**Figure 1A**, **Table 1**). Thus, lung *Mtb* burden distinguishes the more severely infected animals, ATB and *Mtb*/SIV, from less severe, latent and naïve groups. While lung CFU is a clear measure of latent or active TB disease state, it requires extensive sampling of the lung tissue that is generally only possible post-mortem.

**Table 1:** Significant Welch’s t Test Comparisons. Green denotes statistically significant comparisons. We tabulated the total number of significant Figures when comparing between cohorts and found the most differences between ATB and LTBI. (P value of ≤ 0.01).

	CTRL v ATB	CTRL v LTBI	CTRL v *Mtb*/SIV	ATB v LTBI	ATB v *Mtb*/SIV	LTBI v *Mtb*/SIV
**CFU-endpt**	**0.002**	0.374	**0.005**	**0.005**	0.309	**0.012**
**CFU-endptlog_10_**	**0.000**	0.332	**0.000**	0.013	0.726	0.015
**CRP-peak**	**0.000**	0.095	**0.003**	**0.000**	0.250	**0.004**
**CRP-endpt**	**0.001**	0.154	**0.003**	**0.001**	0.203	**0.003**
**A/G-bottom**	NA	NA	NA	**0.005**	0.386	**0.002**
**A/G-endpt**	NA	NA	NA	0.011	0.771	0.012
**Lympho-peak**	NA	NA	NA	0.473	**0.003**	0.042
**Lympho-bottom**	NA	NA	NA	**0.010**	0.158	0.347
**Lympho-endpt**	NA	NA	NA	**0.001**	0.110	0.023
**Mono-peak**	NA	NA	NA	0.412	**0.003**	**0.000**
**Mono-endpt**	NA	NA	NA	0.629	0.074	0.021
**Neutro-peak**	NA	NA	NA	**0.028**	0.222	0.547
**Neutro-bottom**	NA	NA	NA	0.675	**0.003**	0.022
**Neutro-endpt**	NA	NA	NA	**0.002**	0.247	0.021
**VKORC1**	0.096	0.162	0.158	0.075	0.039	0.964
**MGC13114**	0.076	0.128	0.117	0.104	0.034	0.801
**MCAM**	0.127	0.439	0.379	**0.009**	0.020	0.755
**BAX**	0.226	0.373	0.349	0.051	0.042	0.813
**MX1**	0.208	0.183	0.299	0.090	0.032	**0.008**
**IFRG28**	0.032	0.025	0.061	0.411	0.018	**0.008**
**G1P2**	0.100	0.053	0.186	0.020	0.311	0.043
**SECTM1**	0.249	0.121	0.267	0.031	0.901	0.076
**CSNK1G2**	0.075	0.044	0.063	0.074	0.502	0.326
**CDC42BPB**	0.050	0.031	0.044	0.157	0.663	0.426
**C9orf156**	0.083	0.036	0.067	0.054	0.631	0.370
**DL4**	0.182	0.098	0.183	0.043	0.968	0.119
**ABCA3**	0.076	0.038	0.053	0.022	0.273	0.389
**CLEC1**	0.073	0.014	0.041	0.013	0.229	0.035
**DJC1**	0.062	0.032	0.066	0.093	0.962	0.286
**C3orf10**	0.036	0.034	0.020	0.895	0.074	0.491
**FLJ20647**	0.079	0.031	0.066	0.012	0.692	0.220
**ELOVL5**	0.095	0.051	0.068	0.035	0.295	0.440
**SIGLEC11**	NA	NA	NA	**0.000**	0.286	0.266
**FLJ10587**	0.084	0.041	0.055	0.022	0.038	0.346
**UTP11L**	0.077	0.047	0.078	0.126	0.970	0.141
**JTV1**	0.084	0.036	0.054	0.014	0.110	0.281
**ACBD3**	0.077	0.037	0.056	0.044	0.390	0.356
**ASAH1**	0.077	0.039	0.051	0.045	0.252	0.499
**FLJ10357**	NA	NA	NA	0.014	0.318	0.171
**STAT5B**	0.057	0.033	0.052	0.101	0.303	0.173
**OSBPL8**	0.066	0.020	**0.031**	**0.005**	0.012	0.227
**Hs.525079**	0.069	0.044	0.069	0.137	0.955	0.152
**DCAMKL1**	**0.006**	**0.001**	**0.003**	0.299	0.245	0.770
**IHH**	0.052	0.211	0.160	0.025	0.121	0.677
**TTC7A**	0.134	0.054	0.233	0.026	0.047	0.697
**ACRV1**	0.098	0.300	0.096	**0.003**	0.916	0.044
**Number of Significant Comparisons**	**7**	**18**	**9**	**28**	**12**	**17**

The highlighted values represent significant p-value.

### Peripheral blood metrics of disease

The measured values of serum CRP levels in macaques during *Mtb* infection agreed with lung bacterial burden. Serum CRP levels serve as a minimally invasive, reliable peripheral marker of experimental *Mtb* infection and TB disease progression in both RM and cynomolgus macaques (10, 15–17, 21–23, 26, 31, 33–36). In line with bacterial burdens, we found that CRP values for ATB and LTBI were significantly different, although the differences in CRP were not significant between LTBI and CTRL (**Figure 1B**, **Table 1**). Serum CRP levels for the ATB and *Mtb*/SIV groups were similar and exhibited no differences, while significant differences were found between *Mtb*/SIV and LTBI. The ATB and *Mtb*/SIV groups exhibited strong, positive correlations to their respective bacterial loads (**Table 2**). These results support CRP at peak and endpoint as a correlate of more severe *Mtb* infection, with previous studies also suggesting that serum A/G ratio may be a viable biomarker of ATB (24), but similar to CRP, other confounding factors could interfere (37) and convolute the interpretation of results.

**Table 2:** Pearson Correlations to CFU/gm(log_10_). All correlations were made between CFU and clinical metrics or genes.

ATB CFU-endpt log_10_	p-value	coefficient	LTBI CFU-endpt log_10_	p-value	coefficient	*Mtb*/SIV CFU-endpt log_10_	p-value	coefficient
**CFU-endpt**	0.002	0.799	**CFU-endpt**	0.0002	0.997	**CFU-endpt**	0.0001	0.963
**CRP-peak**	0.042	0.592	**CRP-peak**			**CRP-peak**	0.002	0.909
**CRP-endpt**	0.036	0.607	**CRP-endpt**			**CRP-endpt**	0.004	0.878
**A/G-pre**			**A/G-pre**	0.034	0.906	**A/G-pre**		
**A/G-peak**			**A/G-peak**	0.048	0.881	**A/G-peak**		
**A/G-endpt**			**A/G-endpt**	0.013	0.952	**A/G-endpt**		
**Mono-peak**			**Mono-peak**			**Mono-peak**	0.005	-0.865
**Mono-endpt**			**Mono-endpt**	0.005	0.973	**Mono-endpt**		
**BAX**			**BAX**			**BAX**	0.036	0.739
**MX1**			**MX1**	0.011	-0.957	**MX1**		
**IFRG28**			**IFRG28**			**IFRG28**		
**ATP5B**			**ATP5B**			**ATP5B**	0.038	-0.735
**CDC42BPB**			**CDC42BPB**			**CDC42BPB**	0.050	-0.707
**ACBD3**			**ACBD3**	0.025	-0.924	**ACBD3**		
**PDGFRA**	0.017	0.696	**PDGFRA**			**PDGFRA**		

Only significant correlations were tabulated (P value of ≤ 0.05).

A significant reduction in A/G ratio, driven by higher serum globulin levels, was observed in animals with ATB and *Mtb*/SIV, compared to the LTBI group (**Figure 1C**, **Table 1**). A/G ratio at pre-infection, peak, and endpoint all correlated positively to bacterial burdens in the lung (**Table 2**). Myeloid cells, particularly macrophages, act as sites of replication for the *Mtb*. Systemic CRP elevation and modulation of A/G ratio is caused by damage to macrophages during the infection process. Differences were also observed in myeloid cell levels in the peripheral blood. For neutrophils, the differences between LTBI, *Mtb*/SIV, and ATB were statistically significant and easily discernable (**Figure 1D**, **Table 1**). In the case of monocytes however, the differences between groups were subtle and statistically significant only between the LTBI and *Mtb/SIV* groups. We found that monocytes were the only cell type in which correlations to lung bacterial burden were significant (**Table 2**). Blood lymphocytes were detected at the highest percentages in the LTBI group compared to statistically significantly lower levels for the *Mtb*/SIV and ATB groups. While monocytes and neutrophil percentages mimicked the pattern exhibited in CRP values and CFU/gm in which LTBI produced significantly less than *Mtb*/SIV and ATB (**Figure 1D**, **Table 1**). Based on these results, we evaluated transcriptional responses in the lungs of the three groups of RMs to further study the unique patterns associated with each group.

### Gene of interest comparison between cohorts

**Figure 2:**
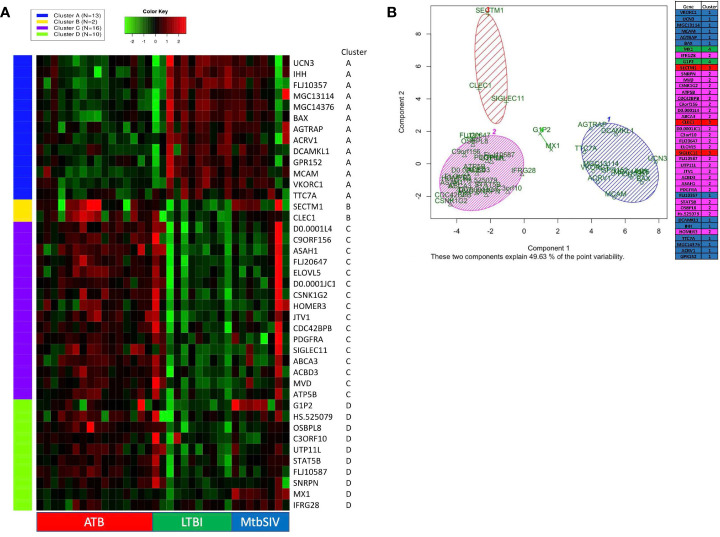
K-means clustering and Principal Component Analysis on 41 GOIs. **(A)** Using iDEP, k-means clustering was performed to identify different clusters in our 41 GOI dataset. The four clusters which were formed (Clusters A-D) are denoted by different colors (Cluster A – Blue, Cluster B – Yellow, Cluster C – Purple, and Cluster D – Green). The gene-expression is denoted by a green-red heatmap with a color key of Green = lower fold=change or repression and Red = higher fold-change or induction, relative to baseline. The key on the right shows Official Gene Symbols, while the key at the bottom shows different samples. Using Graphpad Prism 8, we created a heatmap of 41 genes and animals, with a range of 1e-008 to 6 (red), 0 (black), and -5 to 1e-008 (green) Some RM did not have recorded expression of a particular gene, denoted as white. Out of range values (magenta) were primarily found in LTBI and ATB cohorts, in which values were much lower than the given boundaries of the downregulated gene expression. **(B)** Using R PCA was performed across all cohorts to cluster the 41 genes. A set number of clusters, 4, was formed. Cluster 1 (blue) contained 12 genes, cluster 2 (magenta) contained 23 genes, cluster 3 (red) contained 3 genes, and cluster 4 (green) contained 2 genes. Data is shown for ATB (n=16), LTBI (n=11), and *Mtb*/SIV (n=8).

DNA microarray experiments were performed on RM lung tissue at necropsy, using normal lung as baseline control. To further expand upon differences between gene expression we applied two different clustering algorithms: K-means and PCA. 41 genes were selected as genes of interest through the use of the FSelector package in R. These 41 genes, out of 9800, were best suited to differentiate between the four cohorts. We then performed K-means clustering using iDEP (integrated Differential Expression and Pathway analysis), an application that performs exploratory data analysis, differential expression, and pathway analysis on gene expression data (**Figure 2A**) (27). Four major clusters were identified. Cluster A was defined by a majority of genes with downregulated expression in the ATB, and increased expression in the LTBI groups (**Figure 2A**). The pattern for the *Mtb*/SIV group was mixed with some samples exhibiting ATB- and others LTBI-like pattern. This is not unexpected, since we have unequivocally shown that *Mtb*/SIV co-infected RMs show differential progression after SIV infection – the majority exhibit reactivation, but a minority of animals continue to retain control of infection during the period in which we studied them (1, 16–18, 20, 21). The clusters B-D contained genes with higher expression in ATB relative to LTBI, with two variable outcomes in the *Mtb*/SIV group (**Figure 2A**). Each of these three clusters (B, C, D) however contained unique characteristics. The *Mtb*/SIV group had mostly similar expression of genes in these clusters to LTBI, but with greater variance. Genes like G1P2, MX1 and IFRG28 (Cluster D) exhibited expression patterns that were similar between the ATB and the *Mtb*/SIV groups. Utilizing iDEP we performed a gene enrichment analysis on cluster C, which was the largest cluster, with the majority of genes in it being involved regulation of lipid biosynthesis, storage and localization and steroid metabolic processes (**Table 3**).

**Table 3:** iDEP Gene Enrichment Pathways for K-means clustering.

Cluster	adj.Pval	nGenes	Pathways	Genes
**C**	0.0004	4	Regulation of lipid metabolic process	ELOVL5, ASAH1, PDGFRA, ABCA3
**C**	0.0007	5	Cellular lipid metabolic process	ELOVL5, MVD, ASAH1, PDGFRA, ABCA3
**C**	0.0008	3	Regulation of lipid biosynthetic process	ELOVL5, ASAH1, ABCA3
**C**	0.0009	4	Lipid biosynthetic process	ELOVL5, MVD, ASAH1, ABCA3
**C**	0.0016	3	Steroid metabolic process	MVD, ASAH1, PDGFRA
**C**	0.0038	2	Acyl-CoA metabolic process	MVD, ELOVL5
**C**	0.0038	3	Phospholipid metabolic process	MVD, PDGFRA, ABCA3
**C**	0.0038	6	Phosphorus metabolic process	CSNK1G2, MVD, ELOVL5, CDC42BPB, PDGFRA, ABCA3
**C**	0.0038	4	Organophosphate metabolic process	MVD, ELOVL5, PDGFRA, ABCA3
**C**	0.0045	2	Sphingolipid biosynthetic process	ELOVL5, ASAH1
**D**	0.0029	2	Lipid storage	STAT5B, OSBPL8
**D**	0.0095	2	Lipid localization	OSBPL8, STAT5B

iDEP runs the Parametric Gene Set Enrichment (PAGE) algorithm, which performs one-sample t-tests on the set of genes in the gene ontology biological processes. iDEP identified four different clusters (A–D) in the genes of interest. The adjusted P-values were then used to rank the pathways for each of the principal components.

We also performed principal component analysis (38) using KEGG (https://www.genome.jp/kegg/pathway.html) on the 41 genes of interest (**Figure 2B**). Using Bayesian Information Criterion as part of the mclust package in R, we determined that 4 clusters would be optimal for this dataset PCA in R: http://www.sthda.com/english/articles/31-principal-component-methods-in-r-practical-guide/118-principal-component-analysis-in-r-prcomp-vs-princomp/). Cluster one and cluster two contain the majority of genes. Cluster one contains several genes which are involved in the NADPHoxidase complex and calcium transport and signaling, and one or two genes which are involved in ATP metabolism, apoptosis, and NFkB signaling (**Figure 2B**). Cluster two contains several genes that are heavily involved in cell migration, g-protein reactions, motility, apoptosis, lipid metabolism, and calcium transport. While the larger clusters (cluster 1, 2) contain several genes that serve a myriad of purposes, the smaller clusters (cluster 3,4) have more specialized functionality. Cluster four (green) contains only two genes, G1P2 and MX1, both genes are involved in Type I interferon signaling and antiviral immune response (**Figure 2B**). Both, G1P2 and MX1 are upregulated in *Mtb*/SIV and ATB animals and downregulated in LTBI. Considering the genes strong antiviral activities and the benefit of increased interferon response in more severe infections, as with ATB, it is appropriate that these genes would be upregulated in *Mtb*/SIV and ATB animals (**Figure 2B**). Cluster three (red) contained SECTM1, CLEC1, and SIGLEC11, of the three genes SECTM1 is involved in immune processes and CLEC1 and SIGLEC11 are heavily involved in cell adhesion. Using four clusters only 49.63% of the point variability was explained by the two variables (**Figure 2B**).

**Figure 3:**
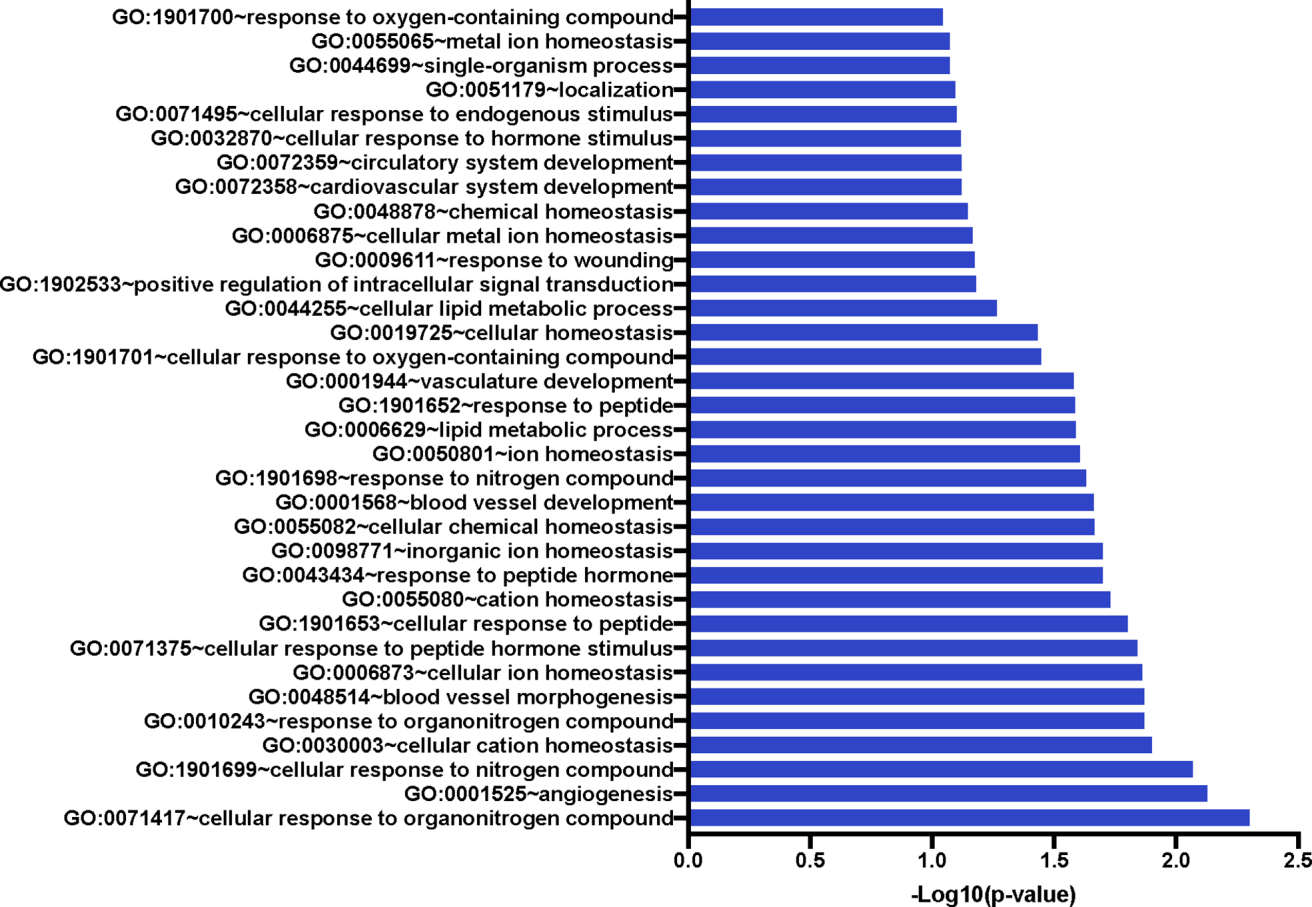
Significant Gene Ontology populated by the 41 genes of interest. The 41 GOIs were used to query DAVID Bioinformatics Resource v 6.8 (https://david.ncifcrf.gov/). We identified numerous GO terms as statistically significant; these included response or cellular response to nitrogen or organonitrogen, as well as response to cellular ion and cation homeostasis. Blood vessel development and morphogenesis terms were also enriched.

We also compared the mean gene expression levels of these 41 genes across the three conditions to identify divergent regulation (**Figure 3**). These genes were significantly enriched in Gene Ontology (GO) terms 0071417 (cellular response to organonitrogen compound), 1901699 (cellular response to nitrogen compound), 0010243 (response to organonitrogen compound), and 1901698 (response to nitrogen compound). The genes populated pathways directly required for the generation of anti-TB immune responses such as NFkB, M1 polarization, ERK/MAPK, and Type I IFN signaling, as well as those pathways that play a role in conditioning cellular or humoral processes indirectly involved in the fine-tuning of immune responses, e.g. Cell adhesion, Calcium Signaling/Transport, Apoptosis, NADPH oxidase, Cell Migration, Cell Proliferation and Antiviral etc., (**Table 4**).

**Table 4:** Gene Pathway of Involvement. Using pathway databases, we identified pathways of involvement for each of the genes.

Pathway of Involvement	Gene
**NFkB signaling**	VKORC1
**M1 polarization**	VKORC1
**Homeostasis**	UCN3
**Cell adhesion**	MCAM, CLEC1, SIGLEC11
**Calcium Signaling/Transport**	MCAM, MX1, FLJ20647, PDGFRA, DCAMKL1, HOMER3
**ERK/MAPK**	MCAM, G1P2, PDGFRA
**Cell proliferation**	AGTRAP, ASAH1
**Apoptosis**	BAX, MX1, JTV1, ASAH1, STAT5B, DCAMKL1
**Type 1 IFN Pathway**	MX1, IFRG28, G1P2, SIGLEC1
**ATP Signaling/Metabolism**	MVD, ATP5B, DNAL4, ABCA3, DCAMKL1
**NADPHoxidase**	ACBD3, FLJ10357, OSBPL8, TTC7A, GPR152
**Cell Migration**	CDC42BPB, C3orf10, DCAMKL1
**Antiviral**	MX1, G1P2
**G protein reactions**	IFRG28, GPR152
**Motility (actin)**	DNAL4, C3orf10
**Lipid Metabolism**	ELOVL5, ACBD3, OSBPL8
**Differentiation**	JTV1, ASAH1, IHH

**Figure 4:**
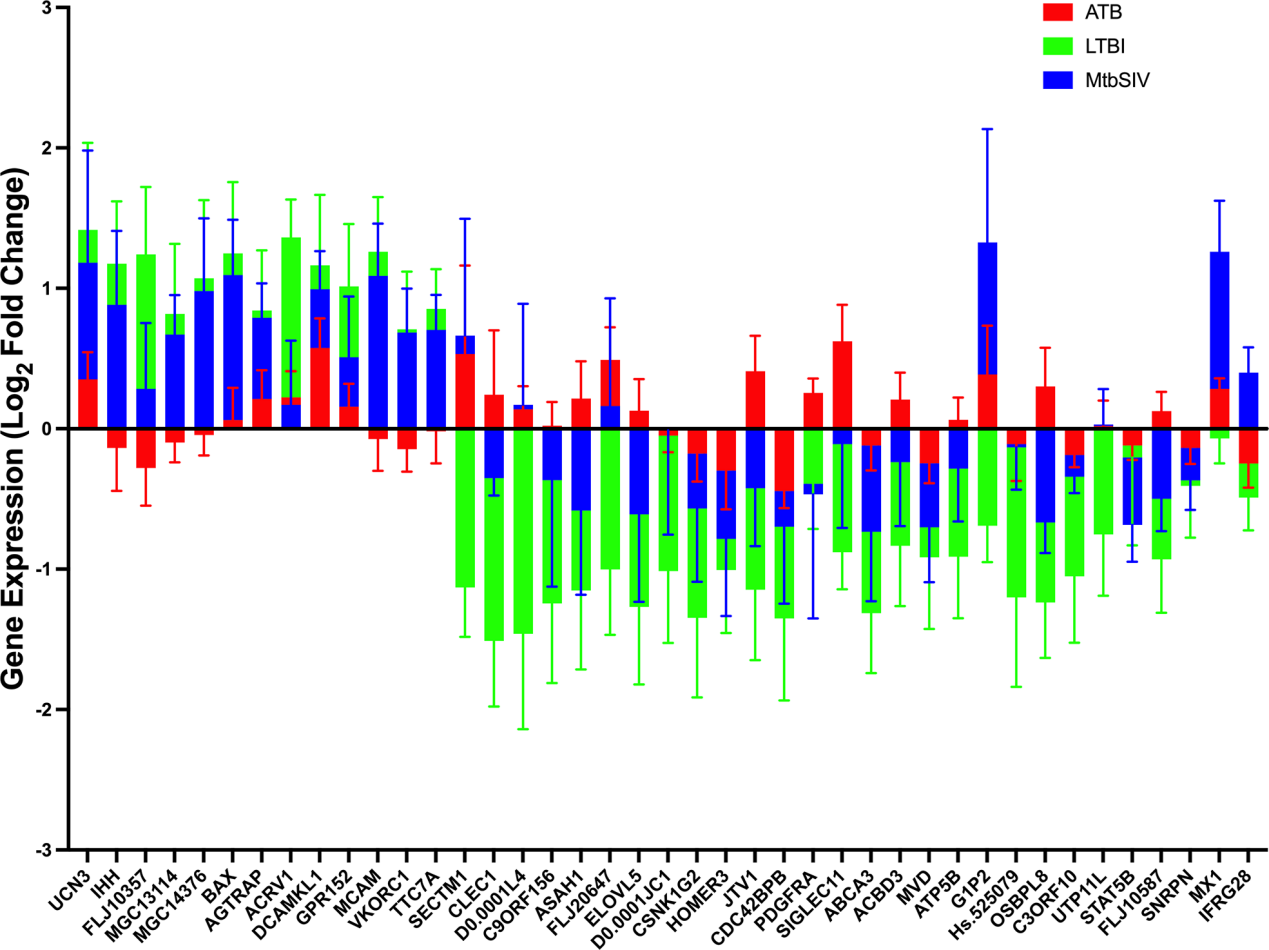
Stacked Mean Gene Expression. As an alternative visualization of the data, mean gene expression ± SEM of ATB (red), LTBI (green), and *Mtb*/SIV (blue) cohorts was plotted.

Next, we studied the genes identified in greater detail. We observed clear differential regulation between LTBI and ATB for DNAL4, ATP5B, CLEC1, FLJ20647, ELOVL5, SIGLEC11, FLJ10587, JTV1, ACBD3, ASAH1, PDGFRA, OSBPL8, with mean gene-expression levels upregulated in ATB and downregulated in LTBI (**Figure 4**). DNAL4 encodes an ATP-dependent molecular motor ATP5B encodes for an ATP synthase subunit; the synchrony in their expression levels being appropriate given both their functions surround the use and synthesis of ATP and progression of *Mtb* infection is characterized by increased cellular movement (The Human Protein Atlas - https://www.proteinatlas.org/) (39). CLEC1, a C type lectin gene, is enriched in several cell types in the myeloid lineage including classical monocytes, intermediate monocytes, myeloid dendritic cells, and is involved in cell-to-cell signaling, cell adhesion, inflammatory response. C type lectin receptors have been closely tied to anti-TB immune responses as pathogen recognition receptors (40). FLJ20647 modulates mitochondrial calcium uptake and cell death pathways, calcium availability in the cytoplasmic area of the cell is vital for NADPH oxidase complex formation and phagocytic clearance of intracellular bacterium (41). Previous studies have positively correlated lipid metabolism and caseous TB granulomas (42); consistent with this ELOVL5, a gene involved in fatty acid metabolic processing, had increased expression in ATB and down regulated in LTBI. SIGLEC11 is involved in anti-inflammatory and immunosuppressive functions, and macrophage, dendritic, and neutrophil infiltration (43). Thus, SIGLEC11 and DNAL4 appear to contribute to cell migration and infiltration to site of infection during TB. SIGLEC1 has also been shown to positively correlate with Type I interferon expression in *Mtb*/HIV coinfected patients (44). The JTVI encoded-protein is a member of the aminoacyl-tRNA synthetase complex, suggesting the need for increased macromolecular synthesis during ATB relative to LTBI (45). The ACBD3 encoded-protein is involved in the maintenance of Golgi structure and recruits PI4K, which is critical for the macrophage response and early T cell activation through CD3 T cell receptor (46). The ASAH1 encoded-protein is involved in the formation of the mature lysosomal enzyme, which catalyzes the degradation of ceramide into sphingosine and free fatty acid. *Mtb* is known to inhibit sphingosine synthesis, as it is required for phagosome maturation (47). PDGFRA is known to play a critical role in lung alveogenesis (48) and injury response in this tissue (49). It is therefore not surprising that the expression of PDGFRA is induced in ATB but not in LTBI. OSBPL8 encodes a lipid transporter involved in the transport of phosphatidylserine and phosphatidylinositol 4-phosphate, the latter being a key constituent of the mycobacterial cell wall target (50).

For a select group of genes, expression was upregulated in LTBI- and downregulated in ATB-samples. These included VKORC1, MGC13114, MCAM, FLJ10357, IHH, TTC7A, and MCG14376. VKORC1 aides in the enzymatic conversion to the active form of vitamin K, and acts in an anti-inflammatory manner through the inhibition of NFkB pathway and macrophage M1 polarization (51, 52). MCAM expressing alveolar macrophages are enriched in the lungs of patients with COPD and it is theorized that MCAM contributes to airway remodeling in chronically obstructed lungs and leukocyte homing (53, 54). FLJ10357 may act as a guanine nucleotide exchange factor to Rho GTPases and aid in the internalization of *Mtb* into the macrophage (55, 56). Similar to MCAM, IHH regulates cell differentiation and tissue remodeling (57). TTC7A, similar to ACBD3, acts as a regulator of PI4K (58). MGC14376 is a hypothetical protein that has been shown to be upregulated during latent cytomegalovirus as well as our latent infection (59).

A subset of genes in both LTBI and ATB cohorts were upregulated, but the magnitude of upregulation differed greatly between groups (**Figures 2A**, **4**). These genes include those encoding for UCN3, AGTRAP, BAX, DCAMKL1, ACRV1 and GPR152. UCN3 is involved in glucose dependent insulin secretion (60). AGTRAP acts as a negative regulator of angiotensin II signaling and has been found to be upregulated in LTBI cohorts in humans in previous meta-analyses of humans gene expression data (61). When host cells are under stress, BAX relocates to the mitochondrial membrane, triggering controlled cell death or apoptosis (62). DCAMKL1 has been associated with microtubule formation and calcium signaling (63), similar to several other differentially regulated genes MCAM, MX1, FLJ20647, PDGFRA, DCAMKL1, HOMER3.

The expression of a family of genes was induced in both ATB and *Mtb*/SIV, but not in LTBI (**Figures 2A**, **4**). These genes encode MX1, G1P2, SECTM1, DNAL4, and FLJ20647. Several of these genes, (MX1, G1P2, SECTM1) can be most notably identified as belonging to interferon response, particularly Type I IFN signaling (**Figures 2A**, **4**). Type I IFN signaling is thought to be detrimental to the host in cases of infection with intracellular bacterium, such as *Mtb* (64). Previous studies indicate that Type I IFN may block hosts ability to limit bacterial replication through upregulation of immunosuppressive pathways (64, 65). It is notable that LTBI cohorts, which exhibit a controlled infection, exhibit a downregulated expression of Type I IFN associated genes, while *Mtb*/*SIV* and ATB cohorts show upregulation in the expression of these genes, indicative of uncontrolled bacterial and viral replication in those groups.

We then studied if genes with significant expression changes between the groups could predict bacterial burden. LTBI had two negative correlations of significance between bacterial burden and the genes MX1 and ACBD3, which are involved in many pathways including antiviral, NADPH oxidase complex, calcium signaling, Type I interferon, and apoptosis (**Table 4**). In *Mtb*/SIV there were two negative correlations to ATP5B and CDC42BPB, which are involved in ATP pathways and cell migration, respectively. *Mtb*/SIV also had one positive correlation to bacterial burden with BAX, an apoptosis relevant gene (**Table 3**). Though the other GOIs did not exhibit significant correlations, that may indicate that there is an indirect effect on bacterial burden rather than a direct one.

**Figure 5:**
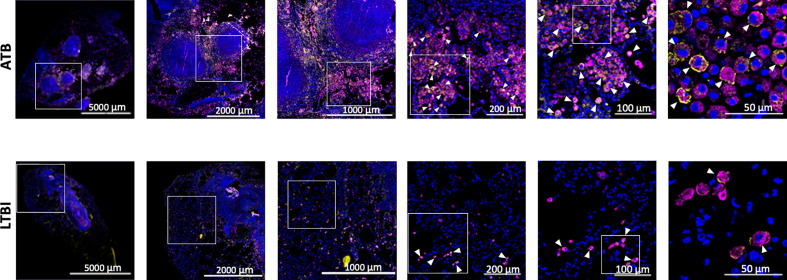
Confocal microscopy based validation of MX1 expression. Multilabel immunofluorescence confocal images validating *in vivo* expression of MX1 (yellow) in CD68 (magenta) expressing macrophage cells in a representative FFPE section from lungs of ATB and LTBI macaques. Arrow heads show macrophages (CD68+) expressing MX1. The images shown here are from the advance stage lesions (granulomas). Majority of the macrophages in ATB lung are seen to express MX1, whereas in LTBI lung, the macrophages expressing MX1 are fewer in numbers.

We used our expertise in immunohistochemistry and multilabel confocal microscopy imaging to validate some findings from our transcriptomics screen. As shown in **Figures 2A**, **4**, several genes involved in Type I IFN signaling were differentially expressed in the lungs of animals with ATB relative to those that exhibited control of infection. Of these we validated the expression of Type I IFN downstream gene MX1 in the lungs of animals with *Mtb* infection that exhibited two different outcomes. The expression of MX1 was detected at significantly higher levels in the ATB relative to the LTBI group, validating the findings from the transcriptomics screen (**Figure 5**). More importantly the expression of MX1 was detected in cells that were positive for the pan-macrophage marker CD68 (macrosialin), a LAMP family glycoprotein. We have traditionally exploited the expression of CD68 in RM lungs to mark macrophages as well as monocytes. In RM lungs CD68 is not expressed on lymphoid or epithelial cells. These results conclusively show that Type I IFN signaling is induced in a *Mtb*-burden-specific manner in lung macrophages during TB disease.

## Discussion

While most people exposed to *Mtb* develop control over infection, there still are more than 10 million reported cases of active TB disease every year, worldwide. Clearly, approaches aimed at identifying those associated with a risk of progression are urgently needed. Previous studies in patient samples from Africa have found Type I IFN signaling to be highly relevant to identifying gene signatures able to differentiate between TB disease states (66). Berry et al. identified 393 gene pattern which allowed them to differentiate between LTBI and ATB patients using whole blood. They were also able to identify 86 genes which differentiate between ATB and other inflammatory diseases (66). Zak et al. identified a 16 gene signature that allowed for the calculation of risk of disease progression in seemingly healthy adolescents (67). Other studies predicted disease progression through the use of a single gene pair, C1q C-chain/T-cell receptor-α variable gene 27, in household contacts with recent exposure (68). Our study is unique in that we have compared control (naïve), ATB, LTBI, and *Mtb*/SIV RM and have sampled from the site of infection in the lung rather than from the peripheral blood. Sampling lung tissue is invasive but this study has reaffirmed that the periphery may not completely predict expression in the local environment of the infection. Through the use of RM, we have employed a controlled system with defined dates of infection and in the absence of treatment. While our results did not identify a direct overlap in the genes identified by Berry et al (66), or Zak et al (67), there was overlap between these data sets at the level of pathways and function. All datasets have extensive contribution from the Type I Interferon pathway and antiviral immune response, including interferon induced genes (IFITM1, IFITM3, IFIT3, IFI44, IFIH1, IFIT3, IFI16, IFI35, IFIT2, GBP1, and GBP2 in (66) and MX1, IFRG28, G1P2, SIGLEC1 in our study. All three datasets are also well represented in other functional categories, e.g., calcium transport and signaling (CACNA1E and CACNA1I in (66) and CAM, FLJ20647, PDGFRA, DCAMKL1, and HOMER3 in our study); apoptosis (CASP4 and CASP5 in (66) and BAX, MX1, JTV1, ASAH1, STAT5B, and DCAMKL1 in our study), etc. It must be kept in mind that our sample was the lung lesion at a defined time after experimental *Mtb* infection, while the human data was obtained from peripheral blood. Furthermore, the 41 GOI set represented those that were able to differentiate between three types of samples: ATB, LTBI and *Mtb*/SIV.

Our results indicate that there is no single gene or metric that is able to fully differentiate between all cohorts; rather it is a cluster of genes coupled with bacterial burden and peripheral markers that allow for an efficient diagnosis. Though there is often phenotypic similarity between ATB and *Mtb*/SIV, LTBI and naïve, on a genetic level those similarities were less apparent to us. A set of 41 genes of interest in concert showed the potential to differentiate between host disease states. We observed a pattern where ATB and *Mtb*/*SIV*, and CTRL and LTBI shared a high level of similarity between themselves (**Figure 1**, **Table 1**). This pattern however, appeared to deviate in regards to gene expression (**Figure 2A**, **Table 1**). These results also suggest that gene-expression during *Mtb* infection and granuloma formation may be characterized by chronic stimulation by reactive nitrogen species and/or other organonitrogen/nitrogen containing compounds such as NO, diamide, azide, hydroxyurea etc.

Our findings suggest that these 41 genes may be useful in differentiating between the three types of cohorts of NHPs exposed to *Mtb*, namely, ATB, LTBI, and *Mtb*/SIV. We believe that our findings are interesting and the gene signature we mention may identify candidate pathways for fruitful investigation in diagnostics and several other clinically relevant areas. The identified genes and their relevant pathways which were differentially regulated in response to infection. A number of those pathways, identified in results section above, were involved in chemotaxis, antiviral activity, Type I interferon, NFκβ signaling, lipid metabolism, calcium signaling, and ATP processing. Macrophages were found to be an important cell type in relation to the differentially expressed genes through M1 polarization pathways, calcium signaling, which plays a critical role in G-protein-coupled reactions in macrophages (69), and NADPH oxidase complex induction, which is necessary for the activation of MAPK/ERK (70) (**Table 3**). Macrophages play an important role in innate immune response to *Mtb* infection; acting as antigen presenting cells to recruit cells to site of infection; activating T cells, and internalizing *Mtb* into the phagosome for destruction though virulence factors (71). Our results therefore suggest that macrophage polarization, activation and their response to key signals such as Type I IFN and Phox may be important events in *Mtb* induced TB disease and reactivation of LTBI in *Mtb/HIV* co-infection. The higher expression of MX1 (**Figure 5**) in ATB, likely on lung macrophages, relative to its lower expression in those animals which exhibited control, could suggest an important, understudied role for Type I IFN signaling in TB. It has been suggested that Type I IFN signaling could both be protective and detrimental in TB (72). Macrophages conditioned with IFN-γ have been considered as M1 or classically activated phagocytes which favor the restriction of *Mtb*. However, alternatively activated macrophages called M2 associated with immunomodulatory and tissue repair functions have also been described in TB and identified in lungs. Type I IFNs have been known to promote anti-inflammatory states in some instances (73), while other reports suggest that this pathway can enhance inflammation (74). IFN-γ signaling, a key protective cytokine produced by activated CD4^+^ T helper cells during *Mtb* infection, has recently been shown to inhibit the expression and function of Type I IFN signaling on macrophages (75). Genes identified to be responsive to both IFN-γ and Type I IFNs are expressed by macrophages in the lungs of RM with ATB. Therefore, our results suggest that the inhibitory effect of IFN-γ on Type I IFN genes may be dysregulated in ATB, leading to the high expression of genes such as MX1 on CD68^+^ macrophages in ATB but not LTBI. Clearly, more information is needed to better understand how the IFN-γ/Type I IFN interplay regulates the ability of macrophages to inhibit *Mtb* replication in lungs, but our results suggest that this interplay may be important.

A drawback of this study is the necessity of lung tissue samples for DNA microarray based profiling. Further analysis is needed comparing these results to that of surrogate lung samples such as bronchoalveolar lavage, or peripheral samples e.g., blood from both NHP and humans, to identify whether these results a translatable to a non-terminal procedure. Of these 41 genes there is the potential, in the future, to further curate the dataset down to a smaller set of genes while still maintaining the capability to differentiate between cohorts. This study can be used as a platform for comparison between differing host disease states and gives a basis of 41 genes which can accurately differentiate between naïve, active, latent, and co-infected TB animals.

## Data availability statement

The data presented in the study are deposited in the Gene Expression Ominbus (GEO), the NCBI microarray database (http://ncbi.nlm.nih.gov/geo/), accession number GSE213850.

## Ethics statement

The animal study was reviewed and approved by Tulane University IACUC committee.

## Author contributions

Conceptualization: DK, SM. Research: MG, DS, BS. Analysis and interpretation: MG, DS, BS, DK, SM. Manuscript writing: MG, DS, SM. Manuscript editing: DS, MG, BS, DK, SM. All authors contributed to the article and approved the submitted version.

## Funding

The work described in this manuscript was supported by NIH awards # R01AI134245, R21AI128130, R21AI127222 and R21AI127160 (to S. M.) and R01AI134240, R01AI111914, and R01AI111943 (to D.K.) and R01AI170197 (to D.K.S.), and by institutional NIH awards P51OD111033 and U42OD010442 to the SNPRC, and P51OD011104 to TNPRC. These funders had no role, however, in the design and execution of the experiments and the interpretation of data. The views expressed here are those of the authors and do not necessarily represent the views or official position of the funding agencies.

## Conflict of interest

The authors declare that the research was conducted in the absence of any commercial or financial relationships that could be construed as a potential conflict of interest.

## Publisher’s note

All claims expressed in this article are solely those of the authors and do not necessarily represent those of their affiliated organizations, or those of the publisher, the editors and the reviewers. Any product that may be evaluated in this article, or claim that may be made by its manufacturer, is not guaranteed or endorsed by the publisher.

## Supplementary material

Supplementary Figure 1 Data is shown for the peripheral blood count for **(A)** Reticulocytes, **(B)** Basophils, **(C)** Eosinophils, **(D)** Red blood cells (RBCs) and **(E)** Platelets compared between all four cohorts, control (black), ATB(red), LTBI (green), and *Mtb*/SIV (blue). Data is shown for ATB (n=16), LTBI (n=11), and *Mtb*/SIV (n=8). Error bars represent Mean ± SEM.
