# Supplementary figures and images for: System-wide identification of myeloid markers of TB disease and HIV-induced reactivation in the macaque model of Mtb infection and Mtb/SIV co-infection
Source: Front Immunol. 2022 Oct 5;13:777733. doi: 10.3389/fimmu.2022.777733 (PMC9583676; doi:10.3389/fimmu.2022.777733)

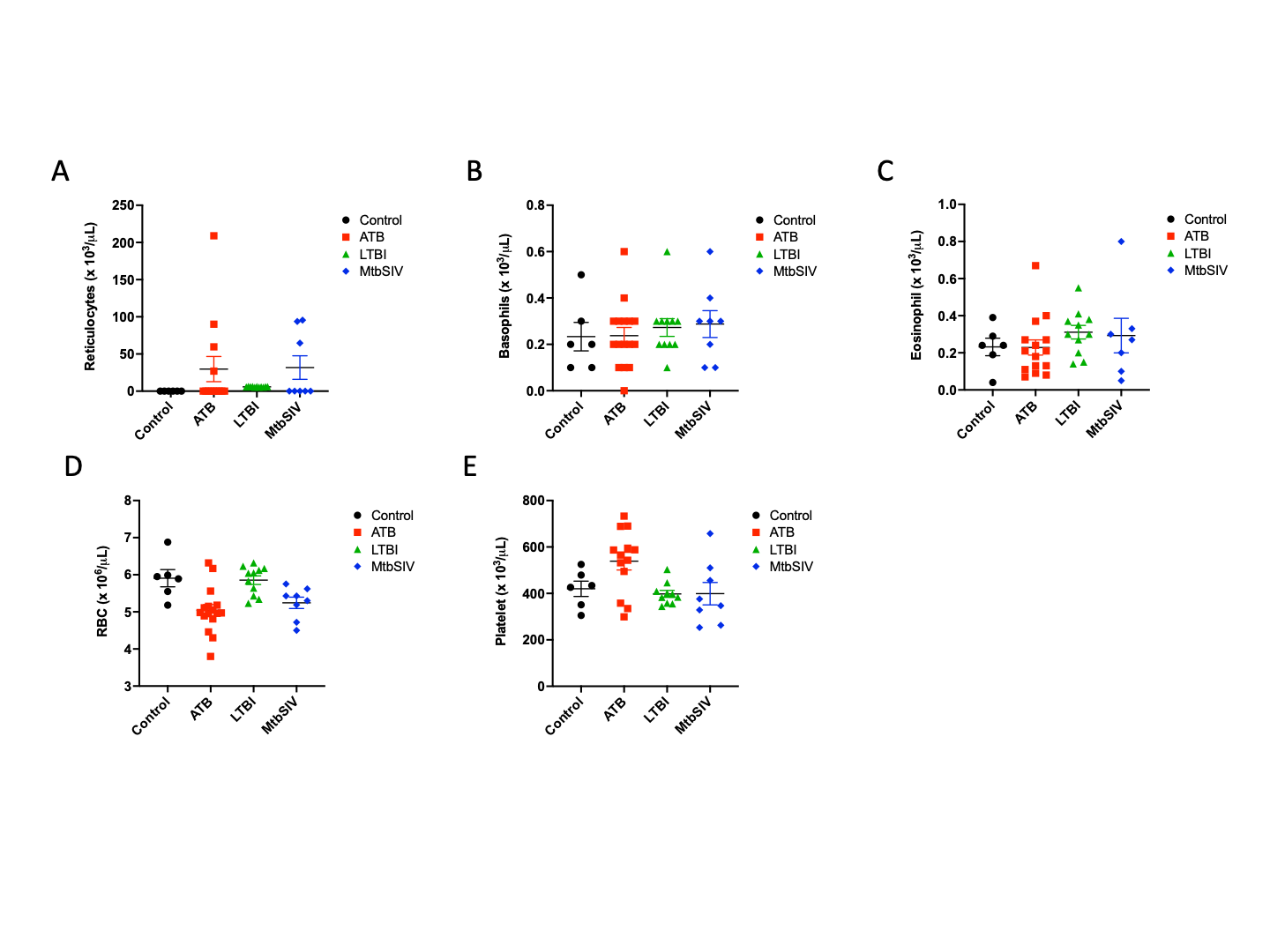

Supplement: Supplementary Figure 1 — Data is shown for the peripheral blood count for (A) Reticulocytes, (B) Basophils, (C) Eosinophils, (D) Red blood cells (RBCs) and (E) Platelets compared between all four cohorts, control (black), ATB(red), LTBI (green), and Mtb/SIV (blue). Data is shown for ATB (n=16), LTBI (n=11), and Mtb/SIV (n=8). Error bars represent Mean ± SEM. [file Image_1.tiff]
